# Supplementary material for: Mechanisms governing the pioneering and redistribution capabilities of the non-classical pioneer PU.1
Source: Nat Commun. 2020 Jan 21;11:402. doi: 10.1038/s41467-019-13960-2 (PMC6972792; doi:10.1038/s41467-019-13960-2)
Supplement: Supplementary file 7 — Source data [file 41467_2019_13960_MOESM7_ESM.zip › Source_Data/FigureS2/FigureS2B_MotifScanOutput/KmeansCluster14/homerResults/motif4.similar.html]

motif4

## Information for motif4

T
C
A
G
T
G
A
C
A
T
C
G
G
T
A
C
A
T
C
G
C
T
A
G
G
C
T
A
G
C
T
A
  
Reverse Opposite:  

C
G
A
T
C
G
A
T
G
A
T
C
A
T
G
C
A
C
T
G
A
T
G
C
A
C
T
G
A
G
T
C
  

|  |  |
| --- | --- |
| p-value: | 1e-34 |
| log p-value: | -7.911e+01 |
| Information Content per bp: | 1.664 |
| Number of Target Sequences with motif | 151.0 |
| Percentage of Target Sequences with motif | 29.61% |
| Number of Background Sequences with motif | 4459.1 |
| Percentage of Background Sequences with motif | 9.85% |
| Average Position of motif in Targets | 99.3 +/- 52.8bp |
| Average Position of motif in Background | 99.4 +/- 79.0bp |
| Strand Bias (log2 ratio + to - strand density) | -0.2 |
| Multiplicity (# of sites on avg that occur together) | 1.08 |
| Motif File: | file (matrix) reverse opposite |

### Similar de novo motifs found

|  |  |  |  |  |  |  |  |
| --- | --- | --- | --- | --- | --- | --- | --- |
| Rank | Match Score | Redundant Motif | P-value | log P-value | % of Targets | % of Background | Motif file |
| 1 | 0.862 | A G C T T A C G A T C G A C G T C G A T A G T C G A T C A C T G A T G C T A G C A C G T A T C G C G T A | 1e-29 | -67.013846 | 19.61% | 5.19% | motif file (matrix) |
